# Supplementary material for: Are carbon emissions trading and green financial instruments synergistic? -Comprehensive quantitative research based on content analysis
Source: PLoS One. 2024 Mar 7;19(3):e0298601. doi: 10.1371/journal.pone.0298601 (PMC10919601; doi:10.1371/journal.pone.0298601)
Supplement: S1 Data — (ZIP) [file pone.0298601.s001.zip › Data/地级市Z_F工作报告.zip!/║╙─╧/╚2├┼╧┐╩╨/└┤╫╘╣1⁄2╓┌║┼í╛┬φ┐╦╩2╛▌═°í┐_2015.docx]

各位代表：

　　现在，我代表市人民政府向大会报告工作，请予审议，并请各位政协委员和其他列席人士提出意见。

　　一、2014年工作回顾

　　2014年，是我们经受考验，克难攻坚，拼搏奋进的一年。面对错综复杂的宏观经济形势和艰巨繁重的改革发展任务，市政府在省委、省政府和市委的坚强领导下，在市人大、市政协的监督支持下，深入贯彻党的十八大和十八届三中、四中全会精神，抢抓《晋陕豫黄河金三角区域合作规划》获批机遇，扎实推进“四大一高”战略，各项事业取得新成绩，转型发展迈出新步伐。全市生产总值1240亿元，增长9.1%；固定资产投资1328亿元，增长19.3%；社会消费品零售总额351亿元，增长12.7%；一般公共预算收入92.5亿元，增长13%；城镇居民人均可支配收入22739元，农民人均纯收入9979元，分别实际增长7%和10.1%。

　　深入实施“四大一高”，转型发展成效显著。坚持“四大一高”引领转型，实施重点项目283个，累计完成投资1025.5亿元，占年计划的186.5%，朝晖铜业1万吨压延铜箔一期等126个项目建成。大通关功能彰显。大通关建设完成投资13亿元，占年计划的169.5%。依托三大“国字号”通关机构，积极实施“属地申报、属地放行”通关模式，4家出口企业享受口岸直通放行待遇，果品、食用菌出口实现新突破。大交通体系完善。全市交通建设完成投资68亿元，占年计划的145%，连霍高速扩建、陕州大道大修等项目建成通车，蒙华铁路项目前期进展顺利。大商贸提档升级。大中海、义乌商贸城等项目经营提升，宏江广场、嘉亿广场等项目稳步推进，万达广场等项目签约落地。大旅游品牌提升。成功举办第20届中国（三门峡）国际黄河文化旅游节，甘山国家森林公园、双龙湾景区成功创建4A级旅游景区，接待国内外游客2374.4万人次，增长15.6%，旅游总收入202亿元，增长14.8%，“黄河三门峡美丽天鹅城”品牌更加响亮。高新产业带动能力增强。全市高新技术产业增加值增长20.3%，是规模以上工业增速的1.9倍；新认定国家级高新技术企业15家，是前十年的总和；新建创新平台15家，总数达到112家；三门峡市产业集聚区创业服务中心被认定为国家级孵化器。事实证明，“四大一高”战略日益成为引领全市转型发展的科学路径和生动实践。

　　持续加快结构调整，经济质量稳步提升。把调结构放在重要位置，打造三门峡经济“升级版”。工业经济稳中提质。加快推进传统产业高端化、新兴产业规模化，规模以上工业实现增加值669亿元，增长10.7%。实施亿元以上工业转型升级项目98个，完成投资247亿元，宏鑫公司1.5万吨铜加工等36个项目建成投产，中金集团黄金产业园等重大项目进展顺利。特色农业效益提升。粮食总产6亿公斤，超额完成省定目标。果品总产22亿公斤，稳居全省首位；烟叶面积和收购量居全省第一；肉类总产11万吨，增幅连续三年居全省首位。规模以上农业龙头企业196家，规模农业示范园区122家，家庭农场220家，市级以上农业产业化集群33个。开发完成“河南省农产品质量安全追溯平台”。建成全省唯一一家国家新型职业农民培育整体推进示范市、全国首批农业信息化进村入户示范市，农民人均纯收入增速居全省第一。现代服务业发展提速。第三产业完成增加值335.2亿元，占GDP的比重比上年提升1.7个百分点。积极发展电子商务，阿里巴巴三门峡产业带上线运营，首批上线企业209家；率先在全省建成农产品电子商务中心。大力发展会展经济，举办各类展会40场，第三届特博会现场销售额5.3亿元。产业集聚区建设升级。全市7个产业集聚区完成固定资产投资620.2亿元，增长25.4%；实现规模以上工业增加值294.7亿元，增长11.3%；带动就业15.6万人，转移农业人口6.3万人。我市在全省产业集聚区观摩中获得小组第一名，4个产业集聚区建成“一星级产业集聚区”，渑池县产业集聚区荣获全省“十快产业集聚区”。节能减排有力有效。对81家企业开展能源审计，依法取缔“三高”企业23家；万元GDP能耗、万元工业增加值能耗在连续六年大幅下降的基础上，分别又下降1.3%和4%，超额完成省定目标。实施减排项目94个，列入国家减排目标的6个项目全部完成。

　　统筹城乡协调发展，新型城镇化扎实推进。新一轮城市总体规划通过专家评审，市新污水处理厂投入运行，完成大岭路等5条城市道路改造。中心城区新增供热面积40万平方米，新增和更新出租车400辆，新开通公交线路2条。上村片区改造一期土地征收完成。“城市管理年”活动深入推进，整治环境卫生，规范车辆停放，市容秩序明显改观。三门峡商务中心区年内完成投资81亿元，“五纵四横”道路网、城市管网和区域电网建成，100栋楼宇封顶，一片现代化新城正在崛起。6个特色商业区在建项目26个，完成投资101.5亿元，渑池县特色商业区荣获全省“十快特色商业区”。新建、改建农村公路385公里，解决20.9万农村居民饮水安全问题。全面启动77个美丽乡村示范村农村环境整治，初步实现了“三无一规范一眼净”。城市融入自然，乡村传承记忆，崤函大地正焕发出勃勃生机。

　　不断深化改革开放，发展活力显著增强。深入推进重点领域改革，持续扩大对外开放，转型之路越走越宽。全面深化各项改革。第一批重点改革事项扎实推进。工商登记制度改革成效明显，全市新登记市场主体1.5万户，增长19.2%。农村土地承包经营权确权颁证试点全面完成。不断扩大对外开放。各类招商活动累计签约项目183个，总投资1000.4亿元。实际利用外资9.6亿美元，增长10.2%；实际到位省外资金301.8亿元，增长15.8%。康耀电子、弘奥生物在“新三板”上市，中原量仪等7家公司进入省上市企业后备队。“耿力GL”商标荣获中国驰名商标，实现了我市中国驰名商标“零”突破。

　　着力保障改善民生，群众得到更多实惠。全市财政民生支出115.8亿元，占一般公共预算支出的70%，省市十项重点民生工程完成年度任务。努力扩大就业。继续实施崤函创业扶持行动，发放小额担保贷款6.1亿元，带动就业2.5万人。加快推进全民技能振兴工程，完成职业技能培训12.3万人次。城镇新增就业6.6万人，城镇登记失业率2.8%。提高社会保障水平。支付各项社会保险资金25.9亿元，城乡低保标准位居全省前列，社会保障的“兜底”功能充分发挥。落实保障性住房建设任务5788套，迎宾花园保障房项目荣获中国人居环境范例奖，让困难群众住上了舒适的房子。加大扶贫开发力度。完成26个贫困村整村推进项目和24个扶贫搬迁安置点建设任务，实现3.07万人稳定脱贫。加快发展社会事业。依托三门峡职业技术学院，与河南科技大学合办本科学院获批；义马市被评为全国义务教育发展基本均衡市。崤函古道石壕段遗址申遗成功，实现入选世界文化遗产“零”突破。三门峡市中心医院建成国家“三级甲等”医院，县级公立医院改革全面启动，100所标准化村卫生室主体全部完工；人口自然增长率4.6‰。2014中国（三门峡）横渡母亲河活动被评为中国体育旅游精品项目。推进生态文明建设。实施“蓝天碧水”工程，持续推进大气、水体、土壤等污染治理，市区环境空气质量优良天数264天，占72.3%。完成人工造林50.2万亩，天鹅湖湿地公园清淤和三期改造提升完成，来三门峡越冬的白天鹅越来越多。积极创新社会治理。预防和化解社会矛盾，维护良好的社会秩序，安全生产、食品安全和信访形势保持平稳，群众安全感居全省第三位。基层民主建设扎实推进，第八届村委会换届选举顺利完成。

　　过去一年，我们按照中央、省委统一部署和市委要求，扎实开展党的群众路线教育实践活动，深入落实中央八项规定和省市实施意见，重点解决“四风”突出问题，有力促进了政风转变。市政府机构改革基本完成，取消和下放行政审批项目45项、具有审批性质的管理事项94项。自觉接受市人大法律监督、市政协民主监督和社会监督，严格执行市人大及其常委会的决议、决定，办理人大代表建议91件、政协委员提案270件。推进依法行政，法治政府建设取得积极进展。强化行政监察和审计监督，反腐倡廉有效推进。精神文明建设成果丰硕，社会主义核心价值观深入人心。国防教育和国防后备力量建设取得新成效，双拥共建活动深入开展，军政军民团结不断巩固，驻峡解放军、武警部队和广大预备役官兵在支持地方经济建设中发挥了重要作用。统计、外侨、民族、宗教、对台、史志、人防、防震减灾、无线电、黄河河务、妇女儿童、残疾人、老龄、慈善等工作取得了新成绩。

　　各位代表，过去一年取得的成绩，离不开省委、省政府和市委的正确领导，得益于方方面面的支持奉献，凝聚着全市人民的辛勤智慧。在此，我代表市人民政府，向全市各行各业的建设者，向各位人大代表、政协委员、各民主党派，向驻峡解放军、武警官兵、公安干警，向广大客商以及关心支持三门峡发展的各界人士，致以崇高的敬意和衷心的感谢！

　　我们既要看到成绩，更要看到前进中的困难和挑战：经济企稳回升基础不牢，部分行业和企业经营困难；结构调整任重道远，增长的动力转换接续不足；资源环境约束加剧，污染治理任务艰巨；公共产品和服务欠账较多，与群众期盼还有差距；政府职能转变和作风建设长效机制需要巩固完善，法治政府建设任务繁重等。对于这些问题，我们不以事艰而不为，不以任重而懈怠，一定高度重视、认真解决，决不辜负全市人民的期望。

　　二、2015年总体要求和主要预期目标

　　今年是深入落实中央“四个全面”战略布局的关键之年，虽然全市经济进入新常态，增速换挡、结构调整、动力转换压力增大，但我们完全有信心、有决心、有能力适应和引领经济新常态。我们的信心和力量，来自于“一带一路”建设、黄河金三角区域合作带来的机遇，来自于市委的坚强领导和“四大一高”战略的引领，来自于我们多年来打下的坚实基础，来自于全市上下明理诚信、善做善成的伟大精神。只要我们紧紧依靠全市人民，就没有克服不了的困难，就一定能够把经济下行的不利影响降到最低程度，就一定能够在经济新常态下取得转型发展的新胜利。

　　今年政府工作的总体要求是：全面贯彻党的十八大和十八届三中、四中全会精神，认真落实市委决策部署，积极适应和引领经济新常态，坚持“求转、求进、求实”总要求，深入实施“四大一高”战略，以提高经济发展质量效益为中心，以转方式、调结构为重点，以传统产业高端化、新兴产业规模化为方向，以全面深化改革、扩大开放合作、强化创新驱动为动力，聚焦转型发展，强化风险防控，加强民生保障，促进社会和谐，全面完成“十二五”规划，努力开创转型发展新局面。

　　2015年经济社会发展的主要预期目标是：在优化结构、提高质量、改善环境基础上，生产总值增长9%；一般公共预算收入增长12%；规模以上工业增加值增长11%；固定资产投资增长17%；社会消费品零售总额增长12%；进出口总额增长8%；实际利用外资增长5%；城镇居民人均可支配收入实际增长8%左右，农民人均纯收入实际增长10%左右；城镇登记失业率控制在4.5%以内；人口自然增长率控制在6.5‰以内；节能减排完成省定任务。

　　各位代表，做好今年的政府工作，要把思想和认识统一到经济进入新常态的重大战略判断上来，按照省委、省政府“一个载体、四个体系、六大基础”的部署，着力扩大增长点，转化拖累点，抓好关键点，稳控风险点，抢占制高点，在积极适应和引领经济新常态中培育新动力、谋求新作为、拓展新空间，努力创造三门峡更加美好的未来。

　　三、2015年重点工作

　　（一）着力深化“四大一高”，增创转型发展新优势。坚持以“四大一高”战略引领经济转型，培育综合竞争优势。

　　抢抓政策机遇，持续提升大通关。落实国务院加快大通关建设的精神，加快申报综合保税区，谋划建设铁路开放口岸和陆港物流中心。落实《晋陕豫黄河金三角区域合作规划》，建立推进机制，梳理政策项目，积极与国家相关部委开展对接，推动尽快出台《河南省关于贯彻落实<晋陕豫黄河金三角区域合作规划>的实施方案》。

　　完善基础支撑，持续推进大交通。加快项目建设进度，确保连霍高速公路改造、三淅高速南段年底前建成通车；争取蒙华铁路、渑垣高速、芮宝高速黄河大桥等项目早日开工；加快推进国道310、209、344线和省道331、245线改造工程。扎实开展干线公路环境综合整治，打造安全、文明、畅通的交通环境。

　　释放消费潜力，持续繁荣大商贸。顺应“互联网+”机遇，大力发展电子商务，持续推动阿里巴巴三门峡产业带建设，培育电子商务示范企业3—5家，加快建设电子商务产业园。大中海、义乌商贸城等项目优化业态、提升管理，增强市场号召力；宏江龙街、嘉亿广场等项目准确定位、集聚品牌，早日开业；万达广场等项目强化服务、明确节点，加快进度。加快建设各县（市）商贸项目，积极发展农产品、果品、建材、汽车等专业市场。办好第四届特博会，以“一节一会”为龙头，大力发展会展经济。抓住“一带一路”建设机遇，加快发展物流业，抓好万石物流、大一物流等项目，谋划推动“一带一路”大宗商品服务中心项目。

　　整合优势资源，持续发展大旅游。落实《三门峡旅游发展三年行动计划》，以沿黄半岛景观带为主体框架，整合天鹅湖、黄河公园、函谷关、地坑院等资源，培育主城区旅游产业增长极。推动双龙湾、黄河丹峡、甘山、燕子山等景区提档升级，完成旅游项目投资25亿元，新增4A级旅游景区2家；办好第21届中国（三门峡）国际黄河文化旅游节。加强宣传营销，重点开拓北京、西安旅游客源市场，接待海内外游客增长13%，旅游综合收入增长12%。

　　实施创新驱动，持续加快高新产业发展。坚持创新驱动“一招上水平”，积极培育战略新兴产业，抓好赛诺维制药、速达电动汽车等项目，加快生物医药、新材料、电子信息等高新技术产业发展；新增高新技术企业5家以上，高新技术产业增加值增长13%以上。新建省市级创新平台10家以上，建好高新企业孵化器，积极创建国家级高新区。

　　（二）着力提高质量效益，主动引领经济新常态。更好地发挥投资的关键作用、消费的基础作用和出口的带动作用，实现经济提质增效升级。

　　强化投资拉动。实施市级重点项目245个，总投资1988.6亿元，年内完成投资600亿元以上。建立“十三五”重大项目库，谋划储备重大项目300个以上。

　　激活消费需求。实施旅游休闲、文化教育、健康养老、节能环保等消费工程，鼓励企业适应个性化、多样化消费潮流，开发新产品、新服务，让大众消费潜力成为拉动经济增长的强劲动力。

　　培育出口比较优势。用好“郑欧班列”机遇，发挥通关比较优势，支持电池隔膜、铜箔、果汁、磨料、香菇等优势出口企业开展技术创新，力争全年新增进出口备案企业50家以上。

　　优化企业服务。加大对规模以上工业企业、限额以上商贸企业、农业龙头企业的服务力度，帮助企业解决生产经营困难。抓好工业经济运行分析，加强煤电油运调节调控，积极拓宽大用户直供、大工业用电渠道，引导产业链上下游企业开展产销对接。加强银企合作，加大对小微企业的支持力度。

　　着力防控风险。坚决制止、严厉打击非法集资活动，妥善化解市场风险、行业风险、金融风险等各类风险，确保经济平稳运行。

　　科学编制“十三五”规划。坚持长短结合，谋划好全局性重大项目、重大工程和重大政策措施，争取一批重大项目、重要试点、重点工作列入国家和省规划。

　　（三）着力优化产业结构，培育持续增长新动力。在稳定增长中优化结构，推动产业结构向中高端迈进。

　　推动工业转型升级。大力发展先进制造业，抓好骏通车辆年产1万辆起重专用车、速达电动汽车、河南能化汽车产业园等项目，加快发展精密量仪、特种专用车、数控机床等终端、高端产品，提升先进制造业比重。改造提升传统产业，实施黄金、煤化工、铝工业等传统产业技改提升工程，帮助企业加快产品结构调整，延长产业链条，让传统产业“老树开出新花”。推进新兴产业规模化，抢抓国家政策机遇，大力发展汽车零部件、新材料、食品药品等产业，抓好灵宝汇源果汁、卢氏乐氏同仁堂药厂等项目建设，让新兴产业“新芽长成大树”。加快“两化”融合发展，实施一批信息化带动工业化项目，开展信息化建设示范企业试点。加快数字化产业集聚区建设，推进信息技术在工业领域的应用。

　　推动特色农业提质增效。粮食产量稳定在5亿公斤；果品产量保持在20亿公斤以上；完成烟叶收购60万担；发展蔬菜5万亩，食用菌1.6亿袋，水产品2万吨。培育现代农业产业集群，加快培育雏鹰生态猪等现代畜牧产业化集群，年内新发展5家市级以上农业产业化集群。抓住新一轮“菜篮子工程”机遇，规划建设一批都市生态农业、现代特色农业示范园区。发展新型农村经济组织，新培育农民专业合作社250家、家庭农场80家，规模以上农业龙头企业达到200家。加强农业基础设施建设，抓好白虎潭水库、大石涧水库等项目，启动总投资2.7亿元的国家抗旱规划项目。解决好农村居民饮水安全问题。抓好精准扶贫开发，集中改善40个贫困村的基础设施，对4400名贫困群众实施搬迁，实现3.7万人稳定脱贫。

　　推动服务业发展壮大。以商务中心区和特色商业区为载体，推动现代物流、金融保险等高成长性服务业提升质量，健康养老、教育培训等新兴服务业扩大规模，商贸流通、房地产等传统服务业增添活力。实施服务业重点项目50个，总投资500亿元以上，年内完成投资180亿元以上，确保第三产业占GDP比重提高1个百分点以上。

　　（四）着力完善科学载体，蓄积产业发展新势能。加快产业集聚区、商务中心区和特色商业区建设，增强战略支撑和综合带动功能。

　　推动产业集聚区“三上一提高”。依托产业集群龙头企业培育工程，实施亿元以上项目50个以上，新开工亿元以上项目20个以上，每个产业集聚区新引进投资5亿元以上项目2个以上。加快污水处理、电力、通信基础设施建设，完善生产生活配套服务，推动产业集聚区持续上规模、上层次、上水平，提高吸引力、竞争力、带动力。

　　推动“两区”繁荣发展。着力壮大产业规模、提升服务能力，年内完成投资160亿元以上。三门峡商务中心区，突出生产性服务功能，年内完成基础设施投资10亿元以上，确保高速公路通道、欢乐谷广场等项目建成投用。持续推进天鹅湖环球金融中心、游客服务中心等一批重点项目，年内新引进投资2亿元以上项目3—5个，尽快形成规模，聚集人气。特色商业区，突出生活性服务功能，年内完成基础设施投资4亿元以上，新引进投资亿元以上项目2个以上。

　　（五）着力推进新型城镇化，构建城乡一体新格局。坚持新型城镇化“一发动全身”，以中心城区和县（市）城区为平台，以农业转移人口市民化为重点，探索城乡一体化发展的体制机制。

　　优化城镇体系和布局。落实《三门峡市科学推进新型城镇化实施方案》，构建空间布局合理、要素配置高效、区域特色鲜明的新型城镇化格局。分步启动中心城区城市建设专项规划编制工作。

　　实施城镇基础设施提升工程。加快中心城区基础设施建设，抓好雨污水管网改造，推进集中供热扩面提质工程。完善城市路网，加快停车场建设；开展城市河流清洁行动，抓好天鹅湖湿地公园、黄河公园、青龙涧河提升工程，健全城市水系、生态廊道、环保设施，坚决治理污染、拥堵等城市病。稳步推进城中村和旧城区改造，加快中心城区上村片区改造进度，每个县（市）、区确定1个以上改造项目，改善人居环境，提升城市品位。

　　持续推进“城市管理年”活动。巩固城市管理成果，完善市容管理标准和办法，健全城市管理长效机制，建成数字化城市监督指挥中心，提升城市精细化管理水平。加强对建筑开发企业的管理，严厉查处违法建设行为，维护城市建设秩序。

　　加快城乡一体化示范区建设。争取《三门峡市城乡一体化示范区规划建设实施方案》早日获批。加快示范区内部基础设施与周边对接。

　　推进农业转移人口市民化。坚持“一基本两牵动三保障”，优先解决进城就业定居的农民工落户。全年转移农村劳动力45万人次，力争年底全市城镇化率达到51%以上。

　　扎实推进美丽乡村建设。编制完成各县（市）、区新农村建设规划，继续抓好农村环境综合整治，保护具有历史文化价值的村落民居，全市美丽乡村示范村达到130个以上，让广大农村更富、更美、更宜居。

　　（六）着力创新体制机制，释放改革惠民新红利。落实国家、省改革措施，努力交出一份为发展加力、让人民受益的改革答卷。

　　深化财税体制改革。推进政府和部门预决算公开，完善政府预算体系，盘活财政存量资金。从严控制一般性支出和“三公”经费。稳步推进扩大“营改增”试点，控制债务规模，化解债务风险。

　　深化农村改革。完成全市农村承包土地确权颁证，新增土地流转面积5万亩。探索建立农村产权流转交易试点，促进交易公开、公正、规范。建立健全集体林权流转合同备案登记制度。支持发展农业保险。

　　深化医药卫生体制改革。全面推进县级公立医院综合改革；深入推进新农合支付制度改革，巩固完善全民医保体系；力争年内完成市直医药流通企业改制。

　　深化其他领域改革。推进国有经营性文化单位转企改制，实施文艺院团股份制改造，支持传统媒体和新媒体融合发展。加快推进企业登记“三证合一”改革，年内力争实现“一证一号”。完成党政机关公务用车制度改革，加快教育、环保等领域的改革。

　　（七）着力扩大开放招商，打造内陆开放新高地。牢牢把握开放招商“一举求多效”，积极融入国家“一带一路”建设，拓展开放空间。

　　突出产业招商。围绕五大千亿级产业集群和优势产业，突出抓好装备制造、电子信息、汽车零部件、黄金珠宝、食品加工等产业招商，围绕产业链上下游引进配套项目。加大现代服务业、社会事业、现代农业和基础设施等领域招商力度，每个县（市）、区新引进投资5亿元以上项目3—5个，确保全市招商签约金额继续保持在1000亿元以上。

　　创新招商方式。推行小分队招商、以商招商等行之有效的做法，更加注重与各类商会、协会和沿海开发区、产业园区合作，抓好长三角、珠三角等重点区域招商，积极承接北京地区产业转移。强化专项督查，促进签约项目落地。

　　积极有序“走出去”。鼓励和引导煤炭、黄金等优势企业“走出去”，在境外建立生产基地。扩大对外工程承包和劳务合作。

　　优化开放环境。坚持优化环境“一优带百通”，完善客商投资项目审批代理制，提高投资贸易便利化水平。建立健全社会信用体系，打造诚实守信的营商环境。

　　（八）着力推动发展绿色化，建设生态文明新家园。推行更加严格的生态保护制度、提倡更加自觉的生态文明行为，建设生态宜居环境，让美丽与发展同行。

　　强力推进节能减排。支持企业采用节能环保工艺技术，坚决淘汰落后产能。加强能源消费、重点污染物排放总量预算管理，健全生态环境补偿机制，推进绿色、循环、低碳发展。

　　铁腕防治环境污染。实施最严格的水资源管理制度，抓好重点流域水污染防治，加强南水北调中线水源地保护，完成省定河流断面水质达标任务。加强工业企业废气治理、黄标车淘汰、城市施工和道路扬尘治理，下大力气改善空气质量。强化畜禽养殖、土壤和重金属污染防治，及时排除环境污染隐患。严格环境执法，对偷排偷放者出重拳，对姑息纵容者严问责。

　　抓好生态建设和修复。实施林业生态提升工程，完成造林25万亩，森林抚育和改造33万亩。扎实推进生态县、乡、村创建，建成绿化模范乡6个、模范村20个。

　　（九）着力保障改善民生，开创幸福和谐新生活。继续实施省市十项重点民生工程，让群众有更多的获得感。

　　努力抓好就业工作。促进“大众创业、万众创新”，服务支持个体工商户发展，全年发放小额担保贷款5亿元以上，确保城镇新增就业4万人以上，下岗失业人员再就业1.8万人以上，动态消除“零就业”家庭，让所有创业者在崤函大地上都能实现价值、成就梦想。

　　完善社会保障体系。城乡居民养老保险基础养老金标准每人每月提高18元，企业退休人员基本养老金再提高10%，稳步推进机关事业单位养老保险制度改革。继续提高城乡低保、农村五保和优抚对象补助标准。加快公租房建设和城市棚户区改造，新建保障房4291套。全面实施临时救助制度，让遇到急、难、特、困的群众受助及时，得到关怀和温暖。

　　办好人民满意的教育。实施第二期学前教育三年行动计划，持续缓解城乡“入园难”问题。完善主城区基础教育布局规划，推进标准化学校建设，逐步消除城镇义务教育“大班额”现象；全面改善农村薄弱学校办学条件，促进义务教育均衡发展。坚持立德树人，积极开展素质教育，不断增强学生的社会责任感、创新精神和实践能力。努力提高职业教育水平，加快职教园区建设，从秋季起免除全日制中职在校学生学费，发挥职业教育在经济发展中的作用。大力发展高等教育，河南科技大学应用工程学院今年开始招生。

　　提升群众健康水平。新农合和城镇居民医保财政补助标准每人再提高60元，完善新农合大病保险，全面推开城镇居民大病保险。加强社区医务人员培训，让群众就近享受优质医疗服务。落实计划生育基本国策，推进免费孕前健康检查城乡全覆盖。扎实开展好全民健身运动。

　　积极繁荣文化事业。以崤函古道为轴线，以仰韶文化遗址等为支撑点，推进“早期中国”文明长廊建设；健全公共文化体系，开展好舞台艺术送农民、政府采购百场戏等文化惠民工程；广泛开展“深入生活、扎根人民”主题实践活动，打造一批优秀文化产品，丰富群众精神生活。

　　创新社会治理。深入推进平安三门峡建设，扎实推进“一村一警”；创新信访工作制度，有效预防和化解社会矛盾。健全完善食品药品监管体制，确保机构、职能、人员、责任“四落实”，提高监管能力，保障群众舌尖上的安全。深化重点领域隐患排查和专项整治，提高应急处置能力，确保安全生产形势持续平稳。

　　继续加强国防动员、国防教育和国防后备力量建设，抓好驻渑部队迁建工作，支持驻峡解放军、武警部队、民兵、预备役和人民防空建设，争创全国双拥模范城“五连冠”。继续做好统计、外侨、对台、民族、宗教、史志、气象、防震减灾等工作。

　　四、加强政府自身建设

　　面对经济新常态、发展新任务、群众新期待，我们将恪守使命、牢记重托，贯彻落实“四个全面”战略布局，深入开展“两转两提”，推进政府治理体系和治理能力现代化，切实增强政府执行力和公信力。

　　扎实推进依法行政。强化法治思维，依法履行职责，做到法无授权不可为、法定职责必须为。推行政府法律顾问制度，深化服务型行政执法，把政府工作纳入法治轨道。认真执行市人大及其常委会决议决定，自觉接受法律和工作监督，主动接受市政协的民主监督，认真办理人大建议和政协提案。广泛听取民主党派、工商联、无党派人士意见，支持工会、共青团、妇联等人民团体发挥作用。深化政务公开，回应社会关切，让群众更好地监督政府，让政府工作更好地体现群众意愿。

　　持续强化务实担当。经济发展进入新常态，精神面貌要有新状态。坚定不移把发展作为第一要务，坚决纠正懒政怠政行为，调速不减势，换挡不松劲，在新常态、新起点上推动新一轮发展。加强执政能力建设，努力克服“换挡焦虑”和“本领恐慌”，不断提高推动科学发展、服务人民群众的本领和能力，善谋善为，善做善成，以良好的工作成效取信于民。

　　不断提高行政效能。加快转变政府职能，深化政府机构和事业单位分类改革，继续取消和下放行政审批事项，全部取消非行政许可审批事项，探索建立政府权力清单、责任清单，改进服务方式，提高行政效能，用政府权力的“减法”，换取市场活力的“乘法”。发扬“钉钉子”精神，抓好重点项目、重点工作的落实，确保全年任务圆满完成。

　　切实加强廉政建设。严格落实廉政建设主体责任，强化“一岗双责”，把廉洁从政贯穿到政府工作的各个环节。完善财政资金支出、工程项目招投标、土地矿产等国有资源交易领域各项制度，切实扎紧制度的笼子。巩固党的群众路线教育实践活动成果，严格遵守中央八项规定和省市实施意见，自觉践行“三严三实”，驰而不息纠正“四风”，推动作风建设常态化。

　　各位代表！

　　新常态蕴含新机遇，新使命呼唤新作为。让我们在省委、省政府和市委的坚强领导下，携手共进，奋勇前行，为加快建设创新开放、富裕文明、平安和谐、生态宜居三门峡而努力奋斗！
